# Comparisons of 3D printed materials for biomedical imaging applications

**DOI:** 10.1080/14686996.2023.2273803

**Published:** 2023-11-14

**Authors:** Mitchell A. Gabalski, Kylie R. Smith, Jeremy Hix, Kurt R. Zinn

**Affiliations:** a Biomedical Engineering, Michigan State University, East Lansing, MI, USA; b Institute for Quantitative Health Science and Engineering, Michigan State University, East Lansing, MI, USA; c Radiology, Michigan State University, East Lansing, MI, USA; d Advanced Molecular Imaging Facility, Institute for Quantitative Health Science and Engineering, Michigan State University, East Lansing, MI, USA; e Small Animal Clinical Sciences, Michigan State University, East Lansing, MI, USA

**Keywords:** Biomedical imaging, 3D printing, prototyping, material science, polymer characterization

## Abstract

In biomedical imaging, it is desirable that custom-made accessories for restraint, anesthesia, and monitoring can be easily cleaned and not interfere with the imaging quality or analyses. With the rise of 3D printing as a form of rapid prototyping or manufacturing for imaging tools and accessories, it is important to understand which printable materials are durable and not likely to interfere with imaging applications. Here, 15 3D printable materials were evaluated for radiodensity, optical properties, simulated wear, and capacity for repeated cleaning and disinfection. Materials that were durable, easily cleaned, and not expected to interfere with CT, PET, or optical imaging applications were identified.

## IMPACT STATEMENT

A guide for selecting 3D printed materials for custom research tools through characterization of their merits and limitations in biomedical imaging.

## GRAPHICAL ABSTRACT

**Figure:**
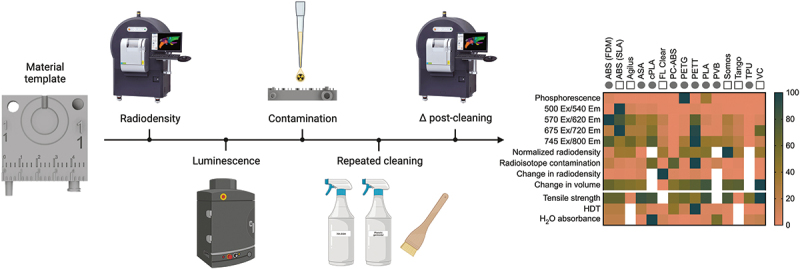

## 1. Introduction

Three-dimensional (3D) printing enables the rapid production of custom tools and is being increasingly utilized for fabrication in the life sciences [1–7]. A range of 3D printers employing different techniques (e.g. fusion deposition modelling, stereolithography, selective laser sintering) are commercially available at varied price points, contributing to the growing ubiquity of 3D printers in research and educational spaces. The library of 3D printable substrates now includes materials that are favorable for a range of applications in healthcare, including those with structural, electrical [1,8], biocompatible [9–12], or bioresorbable [5,13] properties of importance [14]. 3D printing makes for an alluring option for biomedical applications, inexpensive compared to other manufacturing techniques such as injection molding and displaying more dynamic printing capabilities as compared to cast molding and CNC milling. From 3D printed organ-on-a-chip designs [15], to 3D printed bioimplants [16], and 3D printed tissue scaffolds [17,18], 3D printing has become a critical tool in biomedical research.

Preclinical imaging is a rapidly growing research area, accelerating in recent years with the rise of modular and multi-modal imaging instruments. Preclinical imaging refers to the use of biomedical imaging techniques to detect, treat, or monitor disease in animal models before translation to humans. The strengths and limitations of individual imaging modalities [19] may be leveraged to improve data quality by using them in combination, a technique known as multi-modality imaging. Multi-modality imaging may offset the limitations of a particular technique, as is frequently done when computed tomography (CT) data is used to correct positron emission tomography (PET) images for signal lost to attenuation. Alternatively, the combined information provided by multi-modal imaging approaches may improve diagnostic power, as was seen for the detection of head and neck malignancies via PET/CT versus PET or CT alone [20]. Multi-modal imaging may be achieved by manually integrating data acquired on distinct single-modality scanners, or using dedicated multi-modal instruments with integrated or insertable components that enable imaging by two or more techniques.

As researchers pioneer new research spaces, they may find a need for a tool that is not commercially available and thus seek to make it themselves. To this end, 3D printing is frequently used to make these custom tools. Tools 3D printed in-house have been used to manage research subjects during an experiment (for anesthesia [21], maintaining body temperature [22], or monitoring [23]), position samples for imaging (standards [24], phantoms [25], subjects [24,26,27]), and reduce motion artifacts [28]. For example, a 3D printed holder enabled neuroimaging of an awake rat in a clinical MR scanner [29]. Accessories made for multi-modal imaging applications must be compatible with a range of techniques, placing more demands on the construction material and necessitating improved understanding of the properties of the materials used. Thus, it is critical to select the correct polymer for the application and limit interference in the study from introduced artifacts, attenuated signal, or an undesired signal from a responsive polymer.

Desirable characteristics for 3D printed materials for imaging applications include i. no interference with the imaging collection or analyses, ii. lack of toxicity to experimenter and research subjects, iii. rigid and not easily broken, iv. rugged and inexpensive to produce, and v. stability for cleaning using laboratory disinfection protocols. Practical considerations regarding the use of 3D printable polymers in imaging applications, such as the decontamination of an accessory exposed to radioactive material, are often overlooked. Studies have characterized signal intensity (photoacoustic, magnetic resonance imaging), radiodensity (for single-photon emission computed tomography, CT, PET) [30–36], and the impact of ionizing radiation [37] on 3D printed phantoms, but for a limited selection of materials. Less is known about the autofluorescence properties of 3D printed materials, with most fluorescence research focused on sorting select plastics and nano-plastics [38,39] for recycling and toxicology purposes. At this time, the authors are not aware of any resource that guides the selection of 3D printing material for manufacturing of custom tools in a preclinical molecular imaging lab.

This gap was addressed in the current study by characterizing the utility and limitations of 15 3D printed materials within the context of biomedical imaging. The resin and polymer materials were printed from a range of 3D printers and characterized for luminescence, fluorescence, radiodensity, and capacity for repeated cleaning. Additionally, novel information about a 3D printed material’s capacity for decontamination following radiotracer exposure was evaluated. Radioactive contamination is a standing risk in radiology. This report compares and contrasts the performance of common commercial materials in a range of imaging practices, providing the information necessary for manufacturers and researchers alike to choose the best material for their application.

## 2. Methods

### 2.1 Design of template print

**Figure 1.**
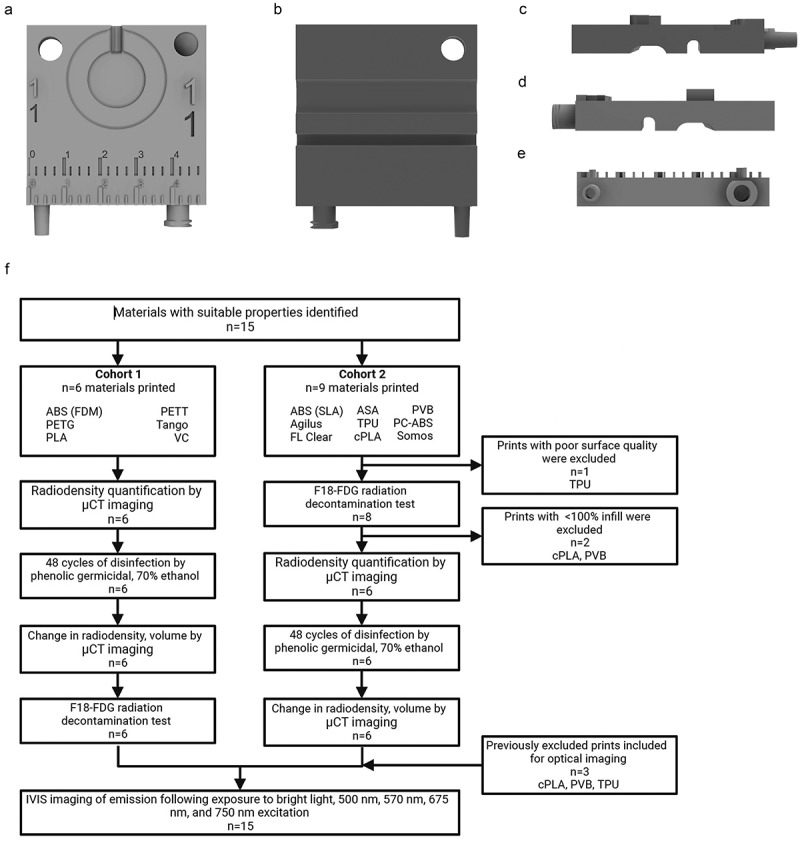
Evaluated materials were printed using from the same template STL file. (a). Front face of template file. High and low relief of numbering in various sizes, rulers, and various connectors are visible. (b). Back face of template file. Grooves for tubing are visible. (c–e). Profile of template print. High reliefs and connectors are visible. (f). Workflow for material evaluations.

A template was designed using Autodesk Fusion 360 that included components common to imaging accessories (Figure 1(a–e)). The template print (5 × 5 × 1.5 cm, L x W x H) included raised and recessed features such as a ruler and standardized air connectors for the former, and a circular breathing pad, valleys for embedded tubing lines, and two threaded screw holes for the latter. Numbers varying in size (2–7 mm) were also included in raised and recessed formats.

### 2.2 Materials

Materials with the following characteristics were prioritized: low density, water resistant, and capacity of withstanding high temperatures. Heat deflection temperature (HDT), tensile strength, and water absorbance values were collected from the literature. Reports that utilized standard industry procedures were sought and supplemented with manufacturer reports or the literature as needed [40–56]. Published data was not found all materials. Selected materials (Table 1) were printed with 100% infill, except for cPLA and PVB.

**Table 1.** Full names and abbreviations for the materials evaluated in this study.

Acronym	Full name	Print method	Location in Figure 2
PETG	Polyethylene terephthalate glycol	FDM	A1
PLA	Polylactic acid	FDM	A2
PC-ABS	Polycarbonate-acrylonitrile butadiene styrene	FDM	A3
PETT	PETT taulman t-glase	FDM	A4
ABS	Acrylonitrile butadiene styrene	FDM	A5
PVB	Polyvinyl butyral	FDM	B1
ASA	Acrylonitrile styrene acrylate	FDM	B2
cPLA	Crystalized polylactic acid	FDM	B3
TPU	Thermoplastic polyurethane	FDM	B4
Somos	Somos watershed XC 11,122	SLA	B5
Tango	Tango	SLA	C1
ABS	Acrylonitrile butadiene styrene	SLA	C2
FL Clear	Clear Formlabs Resin	SLA	C3
VC	VeroClear	SLA	C4
Agilus	Vero + Agilus 30	SLA	C5

### 2.3 Optical emission

Optical emission properties were evaluated for 3D printed materials using the IVIS Spectrum System and Living Image analysis software (Revvity). Phosphorescence was quantified as average radiance (photons/s/cm2/sr) following a photograph flash and 60 second acquisition period with an open emission filter. Material fluorescence was tested independently for each template at commonly used excitation wavelengths (30 nm bandwidth filter), including 500 (GFP), 570 (mCherry), 675 (Cy5.5), and 745 nm (Alexa Fluor 750). Emission filters (20 nm bandwidth) were set to 540, 620, 720, and 800 nm, respectively. Exposure time and binning factor were automatically determined by the instrument to avoid detector saturation; all other camera settings were maintained across materials (high lamp level, 6.6 × 6.6 cm field of view, 1 cm subject height).

The influence of a material’s optical emission properties on signal detection for biological samples was tested. Several liver proteins have autofluorescent properties, producing emissions in the range of 400–500 nm [57]. We excised the liver of a BALB/c nude 194 mouse (Charles River) to identify the excitation/emission wavelength pair that resulted in the highest luminescent image contrast. We tested all non-overlapping filter combinations with 430, 465, and 500 nm excitation and 500, 520, and 540 nm emission peaks. The prevailing combination (465/520 ex/em) was used to capture fluorescence from excised liver, kidney, skin, and bone samples with PETG, PLA, PC-ABS, or the manufacturer-provided Lexan background sheet below. A live BALB/c nude 194 mouse was imaged with the same excitation/emission filters and templates following anesthesia induction and maintenance with 2 and 1.5% isoflurane, respectively, before being recovered. Exposure time and binning factor were automatically determined by the instrument (high lamp level, 13 × 13 cm field of view). All invertebrate procedures were approved by Michigan State University’s Institutional Animal Care and Use Committee.

Image analysis was conducted on luminescent-only images with consistent binning (binning factor 8) and adaptive background subtraction applied. A standardized region of interest (ROI) was used to assess materials imaged independently to mitigate the impact of the shape and thickness of template features on the material’s optical emission profile. ROIs were hand-drawn around biological tissues based on the photograph image. Fluorescence intensity was reported as average radiant efficiency ((photons/s/cm2/sr)/(mW/cm2)) to correct for differences in sample illumination and location in the field of view. Values were plotted in GraphPad Prism.

### 2.4 Radiodensity assessment by microCT imaging

A microCT (μCT) scan was acquired for each material before and after the decontamination procedure using a PerkinElmer QuantumGX microCT scanner (72 mm; 288 µm reconstruction resolution FOV; 8 second scan gantry rotation time x3 overlap stitched; 90kVe/88 uA power setting; Al+Cu x-ray filter; 399 slices). Image analysis was conducted using PMOD 4.2. Radiodensity was quantified by averaging 3 spheres of 3 mm radius and normalizing to the signal of water. Materials that were not printed with 100% infill were excluded. Mean material radiodensity was compared using a one-way ANOVA followed by post-hoc Tukey’s Test in GraphPad Prism software.

### 2.5 Decontamination of radionuclide

A radioactive spill test was conducted to assess each material’s resistance to liquid absorption and contamination. All materials were tested during the same experiment to minimize radiation exposure per ALARA Principles [58] using F-18-FDG acquired from Cardinal Health. Droplets (30 µL) of F-18-fludeoxyglucose (F-18-FDG, *n* = 4/material) were placed in the center of the respiration pad recession on each template print (activity per dose 38.8 µCi). Printed templates were then disinfected with 70% ethanol and dried clean with a Kime wipes after 5 minutes. The radioactivity of each template print was assessed before and after decontamination using a Ludlum Model 3 pancake probe (model 44-9, Serial no. PR059981) and a Capintec Inc. CRC-25 R dose calibrator (Serial no. 113464). The activity on the template print was measured in the bottom of the dose calibrator well for the CRC-25 R and recorded as µCi. For the Ludlum pancake probe, template print activity was measured from the same distance and location each time. Activity was recorded as average counts per minute. Residual activity was calculated as the difference between the pre- and post-contamination measurements after decay-correction to the same timepoint. Mean residual activity was compared for different materials using a one-way ANOVA followed by post-hoc Tukey’s test in GraphPad Prism. One datapoint was determined an outlier and removed by Dixon’s test [59]. Given that material cohorts were at different stages of evaluation when the decontamination test was conducted (see Figure 1(f)), we tested whether repeatedly cleaned 3D-printed templates demonstrated higher residual contamination on average using a two-tailed Welch’s t-test in GraphPad Prism (Supplemental Data File).

### 2.6 Capacity for repeated cleaning

Two months of moderate use was simulated for the 3D printed part, assuming disinfection before and after use 3 times per week. This was done to determine which materials swelled or shrank after being exposed to water and solvent cleaners. The disinfection protocol was selected according to the Center for Disease Control (CDC) standards for the disinfection of smooth, hard surfaces (non-critical items) at healthcare facilities [60,61]. Each round of disinfection included saturation of the surface with a phenolic germicidal detergent solution (Sporicidin, Contec) for 5–10 minutes, scrubbing with a soft bristle brush and water, and saturation of the surface with 70% ethanol. After the decontamination protocol, template prints were imaged by the same methods used previously. Absorption and degradation were assessed following repeated decontamination by comparing the volume of automatically segmented ROIs in pre- and post-decontamination microCT scans, respectively.

### 2.7 Heatmap

**Figure 2.**
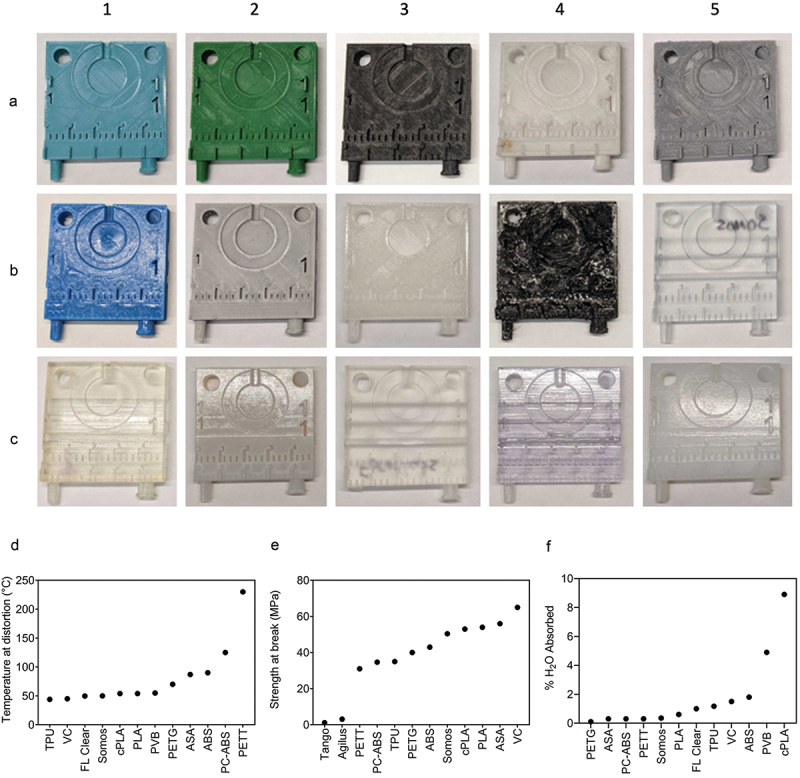
Overview of prints and characteristics for selected materials. A1–C5. Front face image of template, 3D printed from A1. Polyethylene terephthalate glycol (PETG). A2. Polylactic acid (PLA). A3. Polycarbonate-acrylonitrile butadiene styrene (PC-ABS). A4. PETT taulman t-glase (PETT). A5. Acrylonitrile butadiene styrene (ABS) printed via FDM. B1. Polyvinyl butyral (PVB). B2. Acrylonitrile styrene acrylate (ASA). B3. Crystalized polylactic acid (cPLA). B4. Thermoplastic polyurethane (TPU). B5. Somos watershed XC 11,122 (somos). C1. Tango. C2. ABS printed via SLA. C3. Clear formlabs resin (FL clear). C4. VeroClear (VC). C5. Vero + agilus 30 (agilus). D-F. Corresponding heat deflection temperatures (HDT, D), tensile strength (F), water absorbance (F) for selected materials.

**Figure 3.**
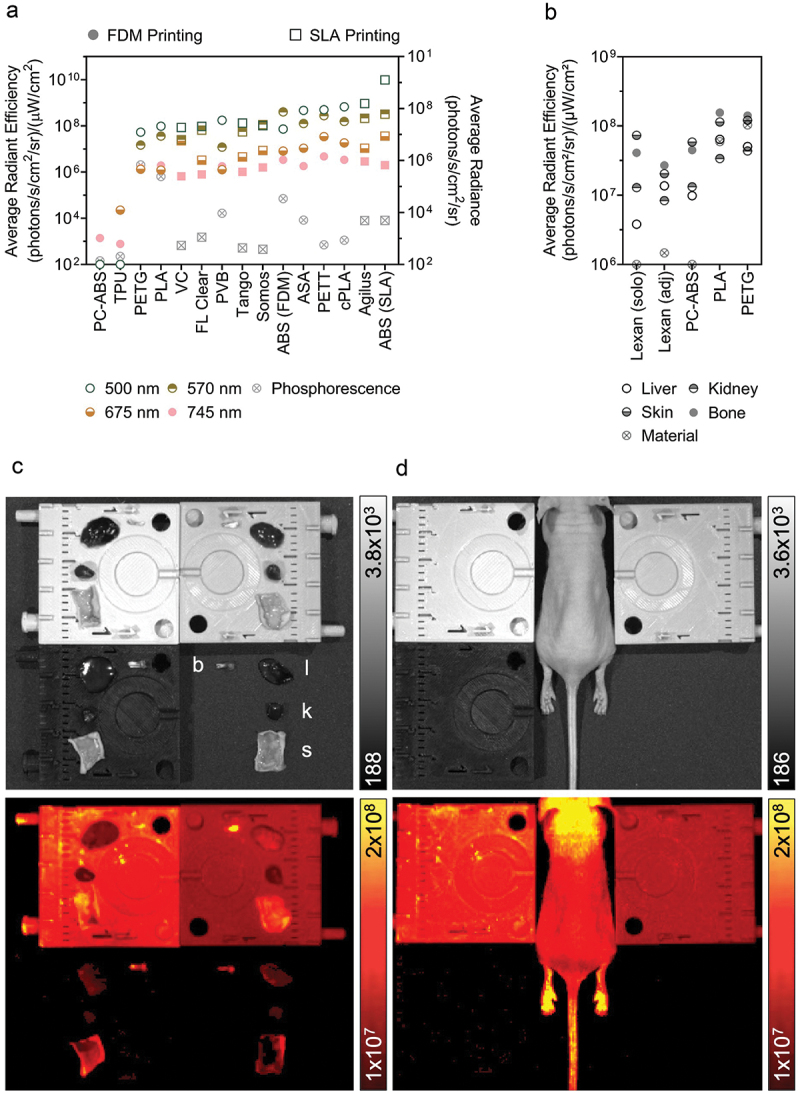
The optical emission profiles of 3D printed materials may interfere in biomedical imaging applications. (a). Phosphorescent and fluorescent signal detected following excitation by wavelengths corresponding to white light (photograph), GFP (500 nm), mCherry (570 nm), Cy5.5 (675 nm), and AlexaFluor 750 (745 nm). (b,c). Quantification of autofluorescence from liver (l), kidney (k), skin (s), and bone (b) samples when imaged alone on lexan (solo; not pictured), adjacent to 3D printed material on lexan (adj; C, bottom right), on top of PETG (C, top left), PLA (C, top right), and PC-ABS (C, bottom left) to illustrate the impact of material fluorescence when imaging biological samples. (d). Naïve mouse imaged between the same samples in C. Fluorescence-only images given in bottom rows of C and D. Emission intensity reported as counts for photographs, average radiance for phosphorescence, and average radiant efficiency for fluorescence.

**Figure 4.**
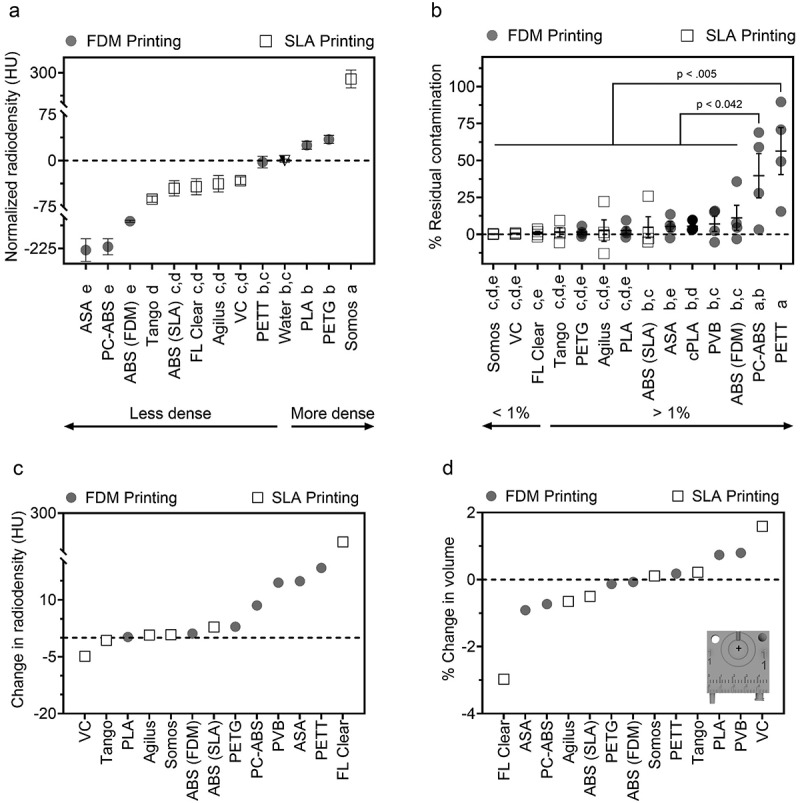
Evaluation of absorption- and degradation-related material risks. (a). Radiodensity of 3D printed templates prior to repeated disinfection by scrubbing, phenolic germicidal, and 70% ethanol (24 cycles). (b). Percentage of residual contamination following exposure to F-18-FDG (droplet placed at +) and subsequent cleaning. (c,d). Change in radiodensity (c) and volume of material (d) following the repeated disinfection protocol described in A. Crosshairs in D indicate location of contamination on all materials. Data given as mean ± SEM. Lowercase letters identify materials with statistically indistinct means.

Heat maps was created to make comparisons easier and allow selection of the best suited material for particular needs. Color values were assigned by normalizing mean values reported in Figures 2–4 to the largest value in that dataset using the batlow color map (40–80% range used, centred on 60%). The batlow scientific color map avoids visual distortion of data and is accessible to readers with color vision deficiencies [62]. White cells indicate a lack of data for that metric and material.

## 3. Results

### 3.1 Design of template and printing of materials

A total of 15 materials were selected for printing and further testing. Their physical properties of interest are summarized in Figure 2. The selected materials were printed via both stereolithography (SLA) and fused deposition modeling (FDM) 3D printing methods. Templates were printed from 5 printers: LulzBot (FDM), Ultimaker S5 (FDM), Polymaker: Polysher (FDM), Formlabs 3B (SLA), and Stratasys J750 (SLA) (Figure 2(a1–c5)).

SLA printers outperformed FDM in print quality due to their ability to print with higher resolution, even down to 2 mm numbers. The 2 mm length numbers were only visible in one FDM print, PC-ABS. The SLA prints also had a smoother surface that lacked print lines inherent to FDM printing. Many FDM prints had frayed edges and lingering support material, which limited fine detailing. Regarding material properties PETT, PC-ABS, and ABS have the highest temperature threshold at distortion (Figure 2(d)), whereas TPU, VC, and FL Clear distort at the lowest temperatures. PETG, PETT and PC-ABS ranked lowest in percent water absorbed (Figure 2(e)), whereas cPLA, PVB and ABS ranked the highest. VC, ASA, and PLA are the materials with the highest tensile strength (Figure 2(f)). Tango and Agilus, being pliable materials, have the lowest strength at break values.

### 3.2 Optical emission properties

All materials demonstrated some degree of emission following excitation with wavelengths commonly used in fluorescent imaging. Tested materials demonstrated a range of emission patterns, including low optical emission overall, primarily high phosphorescence, and primarily high fluorescence (see Supplemental Data File for representative images). Most tested materials displayed little to no phosphorescence, except for FDM ABS, PLA, and PETG with radiance > 10,000 (photons/s/cm^2^/sr). SLA and FDM ABS, Agilus, cPLA, PETT, and ASA were each frequently amongst the top 5 for highest fluorescence intensity per excitation wavelength tested (Figure 3(a)). PLA, FDM ABS, and VC also showed appreciable fluorescence following excitation with 500, 570, and 675 nm wavelengths, respectively. Interestingly, PC-ABS and TPU both had corrected signal intensity values below zero for phosphorescence and excitation at 500 and 570 nm.

Materials that emit light following excitation by wavelengths in the visible and near infrared spectrum may interfere with optical imaging techniques (e.g. fluorescence or bioluminescence). Signal detected from biological samples differed when placed adjacent to or on top of a set of tested materials (PC-ABS, PLA, PETG) relative to samples imaged alone on the standard Lexan sheet. PLA and PETG both fluoresce at intensities similar to the tissues themselves (Figure 3(b–d)), likely resulting in the overestimation of associated tissue autofluorescence due to summed emissions. Further, skin and bone samples were indistinguishable from the PETG background. The background material ROI measured higher for Lexan when imaged in the presence of PLA, PETG, and PC-ABS versus alone. However, lower emission levels were detected from tissue samples in the same region (bottom right quadrant of Figure 3(c)), possibly due to interference from other emission sources (material or tissue). These results demonstrate the risk of erroneous measurements due to signal spillover or interference from luminescent materials when not carefully controlled. Tissue signal was most similar to standard imaging conditions when placed on top of PC-ABS.

### 3.3 Radiodensity assessment by microCT imaging

Materials with high radiodensity are less desirable for nuclear imaging applications (e.g. PET, SPECT) due to expected loss of signal due to attenuation. Materials fell into 1 of 5 groups of statistically non-different means, grouped in the following sets: (a) Somos, (b) PETG, PLA, PETT (equivalent to radiodensity of water), (c) PETT, VC, Agilus, FL Clear, and SLA ABS (equivalent to radiodensity of water), (d) VC, Agilus, FL Clear, and SLA ABS, and (e) comprising FDM ABS, PC-ABS, and ASA. PLA, PETG, and Somos watershed XC 11,122 were the only materials with higher radiodensity than water (Figure 4(a)). ASA, PC-ABS, and FDM ABS were the least radiodense, with all remaining materials falling between fat and water (−50 to 0 HU on CT).

### 3.4 Decontamination of radionuclide

Materials with low residual radioactivity were considered easier to clean and/or more resistant to contamination. PC-ABS and PETT absorbed over 25% the original radiation dose, setting this pair apart from the rest of the panel for risk of contamination. SLA ABS, ASA, cPLA, PVB, and FDM ABS performed similarly to one another, each retaining >4.75% of activity (Figure 4(b)). No statistical differences were observed for the remaining materials (Somos, VC, FL Clear, Tango, PETG, Agilus, PLA), which ranged from 0.19% residual contamination for Somos to 2.6% for PLA. Somos and VC stood apart as having high repeatability relative to other materials. Interestingly, the prints which retained the greatest fraction of radioactivity were overwhelmingly printed via FDM. No difference was found between cohort 1 and cohort 2 residual contamination rates, indicating that 24 cycles of disinfection had no measurable impact on retention of F-18-FDG (Supplemental Data File).

### 3.5 Impact of 2 months simulated use on volume and radiodensity

Following 2 months of simulated use, materials were assessed for change in radiodensity and volume. The greatest change in radiodensity was observed in FL Clear, followed by PETT, ASA and PVB (Figure 4(c)). The remaining materials showed negligible changes in radiodensity. Increase in volume for VC, PLA, and PVB may indicate swelling from water absorption (Figure 4(d)). FL Clear, ASA, and PC-ABS likely experienced degradation as indicated by a reduction in volume, although at low levels (0.16–0.5 ccm difference). Negligible changes to volume were seen in remaining materials following the decontamination protocol.

### 3.6 Heatmap

**Figure 5.**
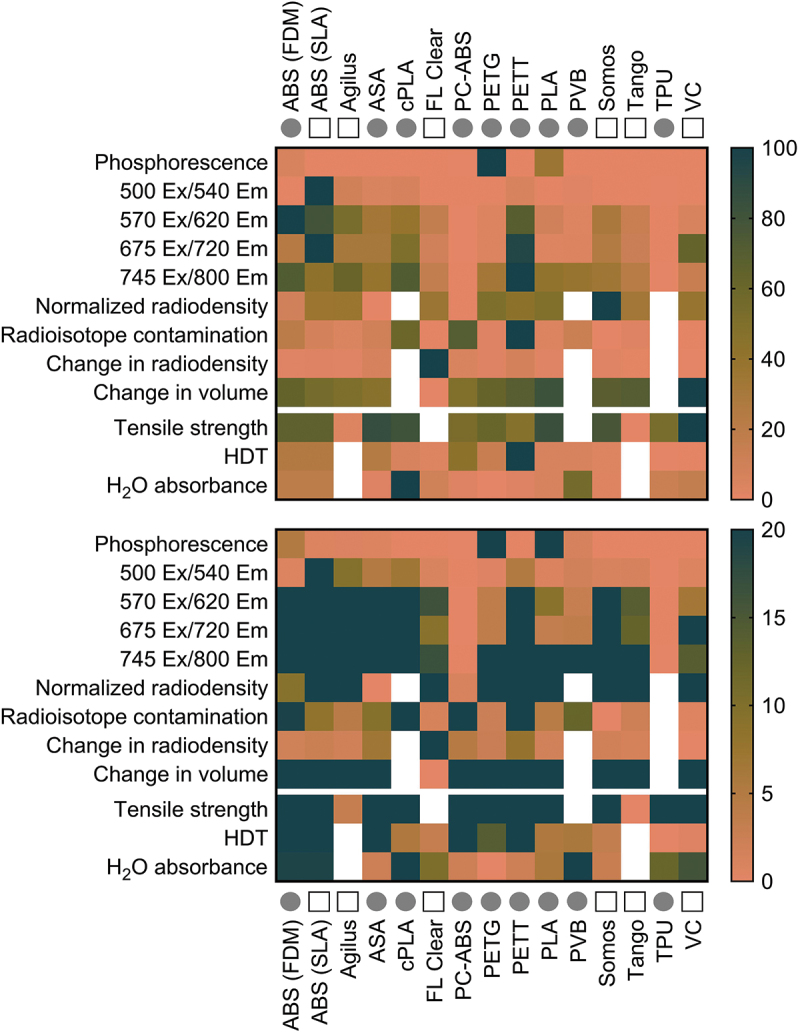
Performance summary for each 3D printed material given in alphabetical order as a percentage of the largest value in each row. The range of values is given in 0–100% (top) and 0–20% (bottom) maximum ranges. Circles denote printing by FDM, squares denote printing by SLA. White cells indicate a lack of data for that metric and material.

The heatmap presented in Figure 5 highlights the range of material performance for our selected metrics, which was wide for some tests and narrow for others. The heatmap also helps to quickly identify materials that stand apart on particular tests. For example, most materials are not of concern for phosphorescence, aside from PETG, PLA, and FDM ABS. Similarly, most materials did not experience major changes in radiodensity values aside from FL Clear. Overall, PC-ABS demonstrated the lowest concern for interference from competing light emission across excitation wavelengths. Materials which quickly absorbed radionuclide are also highlighted (e.g. PETT, PC-ABS, and FDM ABS), and may be avoided for applications where contamination is a concern. With a quick glance, this plot highlights the categories in which the tested materials perform similarly (e.g. phosphorescence, change in volume, radiodensity following decontamination cycles), and those where materials are quite different from each other (e.g. radiodensity, capacity for contamination).

## 4. Discussion

Custom tools are commonly 3D printed by molecular imaging scientists to aid in their research [21–23,26]. However, until now there has been no central resource that compared polymer properties relevant to common molecular imaging techniques. The current study addressed this gap by reporting a variety of metrics that included radiodensity, durability, and optical emission profiles for a panel of 15 common 3D printable substrates. The polymer panel included materials ubiquitous to laboratory spaces (e.g. PETG, PETT, ABS, TPU, and PC-ABS) and 3D printing labs (e.g. PLA, PETG). Routine disinfection methods were applied that included disinfection with 70% ethanol and a germicidal agent. Imaging methods included microCT (QuantumX) and optical methods (IVIS). These studies can serve as a resource for biomedical engineers to efficiently select 3D printed materials that suit their needs from a range of filaments and resins, including many that are commonly used and affordable. Additionally, the testing done here can be applied to any tool made from equivalent materials manufactured from other techniques, such as injection molding, to better understand its characteristics for biomedical imaging.

Each material demonstrated trade-offs for use as a research tool. Thus, materials should be selected for each specific application. For example, Tango performed desirably in all imaging-related tests but has the lowest reported tensile strength of the evaluated materials. Thus, Tango is unlikely to interfere with imaging but was not well suited for applications requiring strength or structure. Similarly, PC-ABS had a low radiodensity and optical emission profile but demonstrated changes following repeated cleaning and rapidly absorbed 40% of applied F-18-FDG. Materials may degrade under some conditions but not others. For example, ASA and PETG both had high deflection temperatures, useful for tools needing to withstand high heat (and/or high pressure such as autoclaving) yet prints from both materials experienced volume changes with repeated use, jeopardizing the long-term use of the material.

This study highlighted the need to carefully select materials for optical imaging accessories. Materials that fluoresce at biologically relevant excitation wavelengths risk interfering with sample emissions and thus data collection. There is a risk of drawing erroneous conclusions from altered emission patterns if the proper precautions are not taken. Contrast between luminescent accessories and imaging subjects or samples will depend on a range of factors, including camera settings, proximity to accessory, autofluorescence of background or sample, and concentration and brightness of light-emitting proteins, if used. Pilot experiments with custom accessories should be conducted in advance of critical experiments to eliminate potential sources of interference and ensure the integrity of collected data. With growing interest in multi-modality imaging and dual-probe imaging reporters, these findings can be applied in the development of animal positioning accessories, multi-modal imaging beds, or other custom tools made from ubiquitous laboratory plastics.

Several materials were identified for their performance in different imaging domains. Materials in the vertical center of the heatmap (Figure 5) may be preferred by optical imaging scientists due to their low response to light excitation, particularly PC-ABS and TPU. Alternatively, researchers interested in making fluorescence imaging makers may benefit from a light-emitting polymer on the heatmap’s vertical left. ABS, Agilus, cPLA, PETT, ASA, PLA, and VC were not recommended for luminescence-focused applications. Regarding nuclear imaging, VC, FL Clear, Tango, PETG, and Agilus demonstrated the lowest level of residual contamination following cleaning and were similarly or less radiodense than water. Of this group, Tango, Agilus, and PETG also experienced little change to volume or radiodensity following repeated cleaning cycles. Thus, these three would make good construction materials for custom tools in nuclear imaging. For microCT applications where contamination is not of concern, ASA, PC-ABS, or FDM ABS may be desirable due to their low radiodensity and thus, low expected signal attenuation. Even Somos (the most radiodense material) was not a significant concern, given that its density was equivalent to soft tissue Hounsfield Unit (HU) values [63]. Regarding mechanical function, tools which will be frequently exposed to liquid but need to retain shape for form and function should be built from PETG, since it has demonstrated low water (Figure 2(f)) and F-18-FDG absorbance (Figure 3(f)). ASA or VeroClear would be well suited for tools needed to withstand high mechanical stress. Overall, FDM printing was a low-cost relative to SLA for printers, materials, and repair. However, this study highlighted risks associated with residual contamination that may inform decision making for applications involving hazardous materials. This trade off may be acceptable due to the low cost of FDM printing filaments, where a contaminated part could be replaced for $0.25/g of material. The SLA resins tested are recommended for applications that require fine details, low risk for contamination, or low radiodensity. No polymer tested performed ideally in every category.

This work could be expanded in several ways. First, one could conduct in vivo imaging with animals using these materials to see how much signal would be attenuated for the various imaging modalities. Second, these 3D printed materials could be further characterized for other applications such as cell culture and capacity to withstand autoclaving. Lastly, branching this work into other fields such as tissue engineering (fluorescent scaffolds) and drug delivery (fluorescent nanoparticles) may be of future interest. Furthermore, the results presented may have utility in the manufacturing of biomedical imaging tools, driving development towards materials with improved performance in the domains that were highlighted.

## 5. Conclusion

In summary, 3D printed materials were identified that were durable and did not interfere with CT, PET, or fluorescence imaging. All materials had low fluorescence at wavelengths greater than 675 nm. SLA prints had the least residual radioactivity after cleaning following a radioactivity exposure event. This information will be useful for biomedical imagers, biomedical engineers, materials scientists, physicists, and manufacturers to improve their understanding of material characteristics and allow selection of the most desirable materials for their 3D printing applications.

## Supplementary Material

Supplemental Material
