# Supplementary material for: Comparisons of 3D printed materials for biomedical imaging applications
Source: Sci Technol Adv Mater. 2023 Nov 14;24(1):2273803. doi: 10.1080/14686996.2023.2273803 (PMC10898812; doi:10.1080/14686996.2023.2273803)
Supplement: Supplemental Material [file TSTA_A_2273803_SM3183.docx]

Comparisons of 3D printed materials for biomedical imaging applications

Supplemental Data

# **Supplemental Methods**

- 1. ***Visualization of varied material emission profiles***

Variation across optical emission profiles was demonstrated by comparing 3 representative materials. Per material, luminescence-only images are stacked in a column for each excitation condition described in section 2.3 of the main text, including the initial photograph.

- 1. ***Corrections to tissue fluorescence***

Ability to correct tissue fluorescence values for samples imaged atop 3D printed materials with fluorescence properties similar to the biological sample was tested by subtracting the average radiant efficiency of the underlying material from the sample. Calculations performed in GraphPad Prism and plotted in the same format as Figure 3b.

- 1. ***Impact of cleaning on F-18-FDG decontamination***

Cohort 1 and cohort 2 differed in test order due to differences in receipt dates (see Figure 1f). We began material characterization while waiting for cohort 2 prints to arrive and conducted. To keep exposure to radiation as low as reasonably achievable (ALARA Principle [https://www.cdc.gov/nceh/radiation/alara.html), the radioactive contamination study was conducted for all materials at once. The order of operations may impact decontamination results is if repeatedly cleaning a template print increases the likelihood of residual contamination following radiation exposure. We tested whether repeatedly cleaned 3D-printed templates demonstrated higher residual contamination on average using a two-tailed Welch’s t-test in GraphPad Prism (Supplemental Data File). Welch’s t-test does not assume equal standard deviation between groups.

# **Supplementary Results**

- 1. ***Visualization of varied material emission profiles***


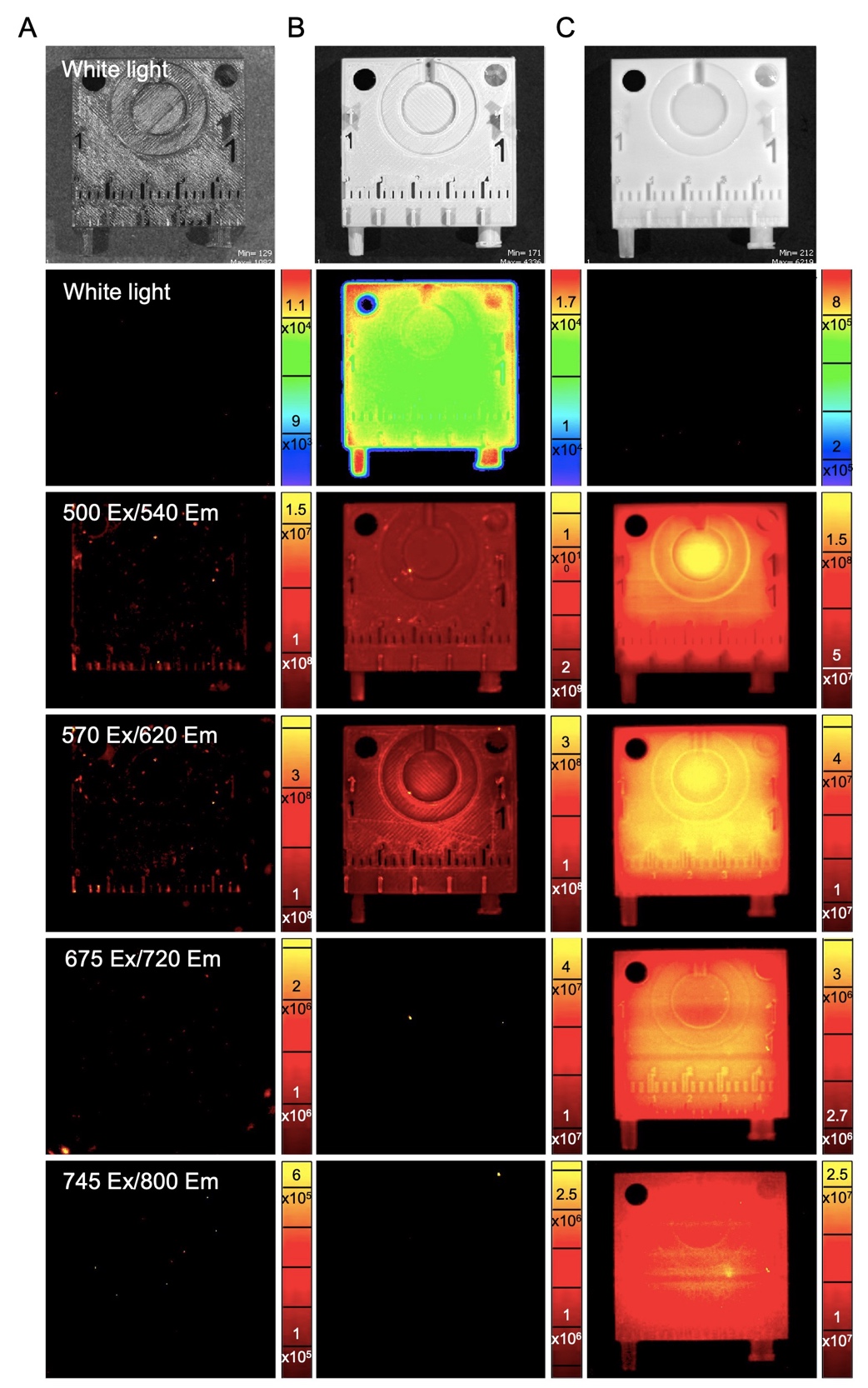


Supplemental Figure 1. Optical emission characteristics of representative 3D printed materials in response to commonly used excitation wavelengths. A. PC-ABS has low emission in all excitation conditions. B. PETG has high phosphorescence intensity and reducing fluorescence with increasing excitation wavelengths. C. SLA ABS demonstrates no phosphorescence but does fluoresce in response to all excitation wavelengths tested. Phosphorescence is shown in units of radiance (photons/s/cm^2^/sr) and fluorescence in radiant efficiency ((photons/s/cm^2^/sr)/(μW/cm^2^)).

- 1. ***Corrections to tissue fluorescence***

Assuming that tissues imaged without 3D printed materials in the field of view is the gold standard, subtraction of material radiant efficiency improved tissue signal quantification for materials that fluoresce at lower levels than the tissue samples (e.g., PC-ABS). For materials that fluoresce at similar magnitude to tissue samples, tissue fluorescence was reported lower than the true value. In some cases, as with the liver and kidney placed on top of PETG, the signal was determined to be less than zero. These results highlight the risk associated with use of materials masking fluorescence from biological samples if the material’s emission properties are not well understood.


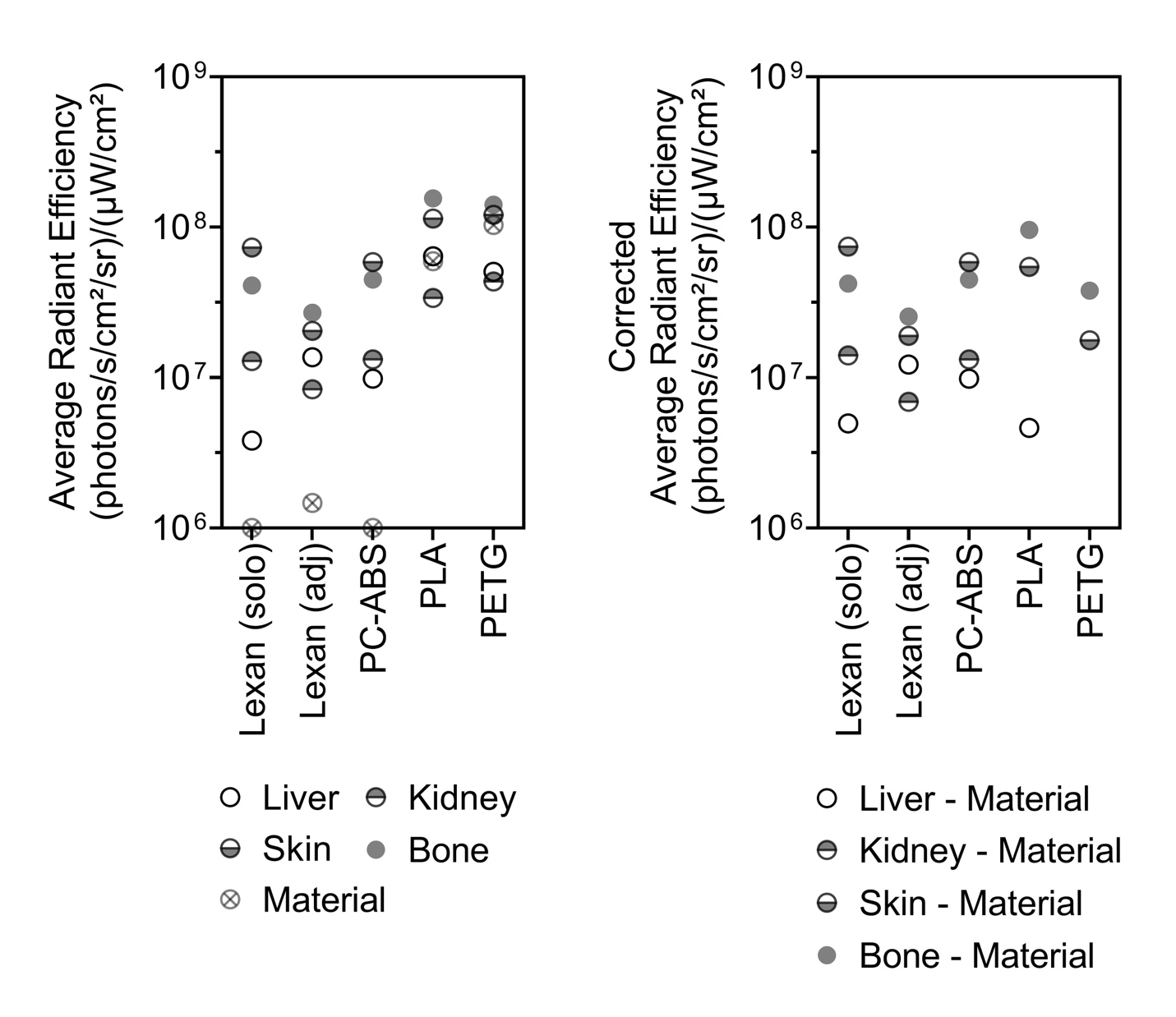


Supplemental Figure 2. Materials with fluorescence intensity similar in magnitude to the tissue of interest are at risk of masking fluorescence from biological samples. The graph from Figure 3B is shown next to the same data with the material average radiant efficiency subtracted from the tissue signal, improving some measurements, and removing others completely (e.g., liver and kidney for PLA, PETG).

- 1. ***Impact of cleaning on F-18-FDG decontamination***

Order of operations did not significantly influence the amount of radiation retained on the print following contamination (p=0.7010, difference between cohort 1 and cohort 2 means = 4.011 +/- 10.060 SEM) or the variability of the results (cohort 1 range 0.5107-56.32, cohort 2 range 0.1894-39.71, p=0.2007 in F test to compare variances). Supplemental Figure 1 gives the mean residual contamination (akin to Figure 4b) and standard error for the two groups.

Supplemental Figure 3. Residual contamination following radiation exposure was not found to be significantly different between cohort 1 and cohort 2 by Welch’s t-test.
